# Comparison between Ceftriaxone and Sulbactam-Ampicillin as Initial Treatment of Community-Acquired Pneumonia: A Systematic Review and Meta-Analysis

**DOI:** 10.3390/antibiotics11101291

**Published:** 2022-09-22

**Authors:** Hideo Kato, Mao Hagihara, Nobuhiro Asai, Jun Hirai, Yuka Yamagishi, Takuya Iwamoto, Hiroshige Mikamo

**Affiliations:** 1 Department of Clinical Infectious Diseases, Aichi Medical University, Nagakute 480-1195, Japan; 2 Department of Pharmacy, Mie University Hospital, Tsu 514-8507, Japan; 3 Department of Clinical Pharmaceutics, Division of Clinical Medical Science, Mie University Graduate School of Medicine, Tsu 514-8507, Japan; 4 Department of Molecular Epidemiology and Biomedical Sciences, Aichi Medical University Hospital, Nagakute 480-1195, Japan

**Keywords:** meta-analysis, ceftriaxone, sulbactam-ampicillin, community-acquired pneumonia

## Abstract

Current guidelines recommend the use of ceftriaxone and sulbactam-ampicillin for the initial treatment of community-acquired pneumonia (CAP). However, there are no clear data on these guidelines. Therefore, this systematic review and meta-analysis aims to evaluate the effectiveness of ceftriaxone and sulbactam-ampicillin in the initial treatment of CAP. The Embase, Scopus, PubMed, Ichushi, and Cumulative Index to Nursing and Allied Health Literature databases were systematically searched from inception to July 2022. The studies included patients who received ceftriaxone or sulbactam-ampicillin as the initial antibiotic therapy for CAP. The mortality and clinical cure rates were evaluated. Of the 2152 citations identified for screening, four studies were included. Results of the pooled analysis indicated no significant differences in the mortality and clinical cure rates between patients treated with ceftriaxone and those treated with sulbactam-ampicillin (mortality, odds ratio [OR]: 1.85, 95% confidence interval [CI]: 0.57–5.96; clinical cure rate, OR: 1.08, 95% CI: 0.18–6.44). This study supports the guidelines for CAP treatment, though further studies are needed to obtain a deeper understanding.

## 1. Introduction

Community-acquired pneumonia (CAP) is an infectious inflammation of the lung parenchyma and remains an important disease threatening human health. The overall incidence rate of CAP ranges from 1.07 to 14 per 1000 persons/year [1,2,3]. In the United States, CAP accounts for more than 4.5 million outpatients and emergency room visitors [4]. Moreover, the annual total cost of treating CAP is USD 250 million [5]. Currently, CAP treatment is faced with several problems and challenges associated with high mortality and economic burden [5,6].

CAP is primarily treated with antibiotic therapy. The appropriate selection of antibiotics at the early stage of infection is key to improving the efficacy of therapy. Initial antibiotics are empirically selected on the basis of pathogen distribution and antimicrobial resistance. *Streptococcus pneumoniae* and *Haemophilus influenzae* are commonly isolated from patients with CAP in many countries [7]. However, the identification of causative pathogens is difficult in patients with CAP, and the percentage has been reported as 40% [8]. Therefore, the optimal antibiotic for CAP treatment is still unclear.

Various guidelines recommend ceftriaxone and sulbactam-ampicillin as first-line antibiotics for CAP [9,10,11]. Ceftriaxone has a spectrum of activity against microorganisms, which are the predominant pathogens in aspiration pneumonia, that is similar to that of sulbactam-ampicillin [12]; meanwhile, ceftriaxone does not target the full spectrum of oral anaerobes that cause aspiration pneumonia [13,14].

Most patients with CAP are treated for mild CAP at the outpatient and primary care clinics [15]. Ceftriaxone has been reported as the most commonly used antibiotic for CAP treatment because of its less frequent administration, lack of requirement for initial dose adjustment according to renal impaired functions, and status as an alternative therapy for patients who are allergic to penicillin [16,17]. However, clinical efficacy of ceftriaxone for CAP treatment is controversial because of the limited data.

Only one meta-analysis has provided evidence of the effectiveness of antibiotics in the treatment of CAP [18]. However, this meta-analysis did not include studies that compared ceftriaxone with sulbactam-ampicillin. Thus, it remains unclear whether ceftriaxone or sulbactam-ampicillin is a more effective treatment for CAP. To date, three retrospective studies have compared the efficacy of ceftriaxone with that of sulbactam-ampicillin in the treatment of CAP [19,20,21]. Moreover, a prospective study was recently published [22]. 

Hence, this systematic review and meta-analysis aimed to evaluate the efficacy of ceftriaxone and sulbactam-ampicillin as initial treatment for CAP.

## 2. Results

### 2.1. Systematic Review

**Figure 1:**
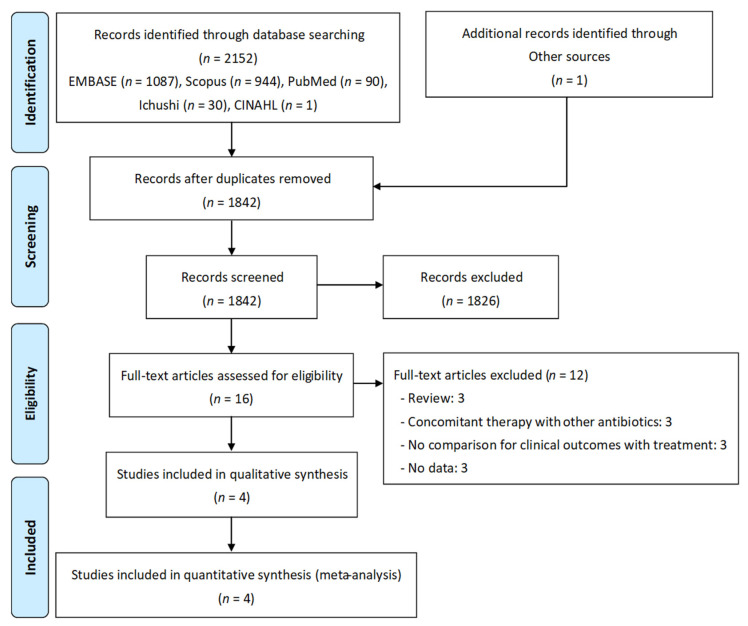
PRISMA flow diagram of the study selection process.

The systematic review of electronic databases resulted in the identification of 2152 articles. After reviewing the titles and abstracts, 1842 articles were deemed ineligible. A full-text review of 16 articles was performed. Figure 1 shows the full list of exclusion criteria. Eventually, four studies met our inclusion criteria [19,20,21,22].

The characteristics of the four studies are summarized in Table 1. All included studies were conducted in adult Japanese patients. Three were retrospective studies [19,20,21], while one was a randomized controlled trial (RCT) [22]. All included studies, except for one, were conducted in a single center [21]. Two studies reported pneumonia mainly due to *S. pneumoniae* and *H. influenzae* [21,22], while the others did not report the bacteriological origins [19,20].

The risks of bias in the assessment results are presented in Table 2. The risks of bias regarding the selection of participants, measurement of exposure, incomplete outcome data, and selective outcome reporting in all studies were relatively low. The confounding variables in the study reported by Shinoda showed a high risk of bias [19]. As the study reported by Hamano was an RCT, the risk of bias was low for all items [22].

### 2.2. Meta-Analysis

**Figure 2:**
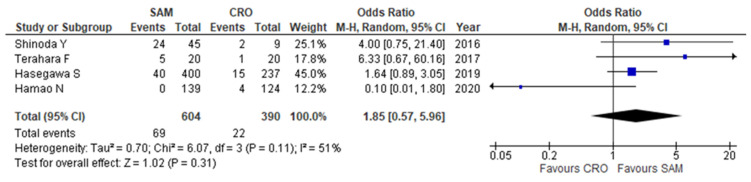
Forest plot presenting the odds ratios for mortality in patients treated with sulbactam-ampicillin and those treated with ceftriaxone in community-acquired pneumonia. CI, confidence interval; CRO, ceftriaxone; M-H, Mantel-Haenszel; SAM, sulbactam-ampicillin; blue square, OR of each study; Black rhombus, pooled OR.

The mortality rates extracted from the four studies were 5.6% (22/390) for patients receiving ceftriaxone and 11.4% (69/604) for those receiving sulbactam-ampicillin [19,20,21,22]. The mortality showed no significant difference between ceftriaxone and sulbactam-ampicillin (odds ratio (OR) 1.85, 95%, confidence interval (CI): 0.57–5.96, I^2^ = 51%, Figure 2).

**Figure 3:**
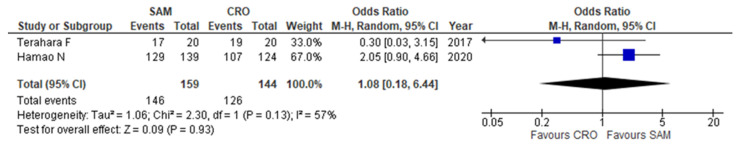
Forest plot presenting the odds ratios for clinical cure rates between sulbactam-ampicillin and ceftriaxone in community-acquired pneumonia. CI, confidence interval; CRO, ceftriaxone; M-H, Mantel-Haenszel; SAM, sulbactam-ampicillin; blue square, OR of each study; black rhombus, pooled OR.

The clinical cure rates extracted from two studies [20,22] were 87.5% (126/144) for patients receiving ceftriaxone and 91.8% (146/159) for those receiving sulbactam-ampicillin. The clinical cure rate was comparable between ceftriaxone and sulbactam-ampicillin (OR 1.08, 95% CI: 0.18–6.44, I^2^ = 57%, Figure 3).

## 3. Discussion

The present meta-analysis showed no significant difference in the incidence of mortality between patients receiving ceftriaxone and those receiving sulbactam-ampicillin. The clinical cure rate also showed no significant difference between the two antibiotics. In addition, no significant difference was found between ceftriaxone and β-lactam/β-lactamase inhibitor combinations in the treatment of CAP [23,24], which is consistent with our findings. Preliminary meta-analysis with limited data shows no difference in clinical effectiveness. Although the present evidence supports guidelines for the treatment of CAP, more RCT studies are needed to obtain a deeper understanding.

Clinically, 40–60% of the patients with CAP have an unidentified pathogen despite performing bacteriological tests [8], and it remains unclear whether initial treatment is crucial based on the guidelines. Although previous guidelines have recommended β-lactam/β-lactamase inhibitor combinations, especially sulbactam-ampicillin, for the initial treatment of CAP [25,26], current guidelines have added ceftriaxone as the initial antibiotic for CAP [9,10,11]. Therefore, our findings provide robust evidence to support these guidelines for CAP treatment.

The differences in effectiveness between ceftriaxone and sulbactam-ampicillin in the treatment of CAP could be attributed to the anaerobic bacteria, which are common causes of aspiration pneumonia [27]. However, anaerobic bacteria can also cause pneumonia in patients without any apparent risk factors for aspiration pneumonia. Molecular methods using 16S ribosomal ribonucleic acid gene sequences have yielded the bacteriological information of CAP and demonstrated that anaerobes were detected in 17.9% of patients with CAP [28]. As mentioned in this study, it is not clear whether anaerobic bacteria are the primary cause. In fact, the included studies did not completely report the causative pathogens.

*S. pneumoniae* and *H. influenzae* are the two most common aerobic isolates associated with CAP [29]. Ceftriaxone is superior to sulbactam-ampicillin for the treatment of penicillin-resistant pneumococci based on the clinical efficacy and in vitro susceptibility [30]. Additionally, ceftriaxone has superior in vitro activity compared with sulbactam-ampicillin against *H. influenzae*, including ampicillin-resistant strains [31]. Therefore, ceftriaxone will likely reduce the mortality rate to a greater degree compared with sulbactam-ampicillin. By contrast, ceftriaxone targets a narrower spectrum of anaerobes associated with CAP compared with sulbactam-ampicillin [12] and has low susceptibility to *Prevotella* spp., which are the primary oral anaerobes associated with CAP [32,33]. Moreover, a recent study reported that β-lactamase-positive strains were detected in 80–85% of ceftriaxone-susceptible anaerobes [34]. Therefore, considering anaerobic bacteria and β-lactamase-positive strains as causes of CAP, sulbactam-ampicillin will more likely improve the mortality and clinical cure rates in patients with this condition compared with ceftriaxone. In particular, it is necessary to consider sulbactam-ampicillin in patients with an unfavorable clinical course with ceftriaxone therapy. However, further studies are needed to associate the effectiveness of antibiotics with CAP pathogens.

Our meta-analysis has some limitations, the most important of which is the lack of sufficient data. In fact, retrospective studies investigating the efficacy of CAP treatment were primarily included in this meta-analysis. Therefore, they are susceptible to bias and confounding factors. However, a prospective study was included in this study. Moreover, more in-depth analyses were precluded, as individual patient data were not available. Finally, the bacteriological origin of CAP was not identified in detail. Therefore, further well-designed studies are required to confirm our findings.

## 4. Materials and Methods

### 4.1. Study Design, Data Sources, and Search Strategy

This study was conducted according to the Preferred Reporting Items for Systematic Review and Meta-Analysis guidelines on reporting systematic reviews and meta-analyses (Figure S1) [35,36]. The following population, intervention, comparison, and outcomes criteria were used for study selection: population (P), patients with CAP; intervention (I), patients treated with ceftriaxone; comparison (C), patients treated with sulbactam-ampicillin; and outcome (O), overall mortality and clinical cure rate. The Embase, Scopus, PubMed, Ichushi, and Cumulative Index to Nursing and Allied Health Literature databases were searched from inception to 26 July 2022 using a combination of the following terms: “pneumonia,” “ceftriaxone,” and “ampicillin sulbactam.” If the original article did not include enough information about the outcomes, we requested additional data from the corresponding authors through e-mail. Only articles published in either English or Japanese were included.

### 4.2. Study Selection

Two reviewers (HK and MH) independently screened the publications based on the titles and abstracts, and subsequently evaluated the full text of the candidate articles. The articles were reviewed based on the inclusion and exclusion criteria, and studies were identified for final qualitative synthesis and meta-analysis. If the original article did not include sufficient information about the outcomes, additional data were requested from the corresponding authors through email. One author (HM) resolved any disagreement between the two primary reviewers [37].

### 4.3. Eligibility Criteria

Studies that met the following criteria were extracted: (i) RCTs and retrospective observational and cohort studies, (ii) studies conducted on patients diagnosed with pneumonia, and (iii) studies conducted on patients receiving ceftriaxone or sulbactam-ampicillin as an initial treatment. Studies that included patients with lung abscesses were excluded.

### 4.4. Data Extraction and Risk of Bias Assessment

The individual study data were extracted using a standardized data extraction form. The data included study design, setting, period and country of the study, total number of participants, agents used along with the dosage, number of patients included, duration of therapy, age of patients, population, and isolated pathogens. The primary outcome was all-cause mortality. The secondary outcome was clinical cure, which was defined as the resolution of baseline clinical signs and symptoms of pneumonia with improvement of the patient’s condition [38]. The risk of bias was assessed independently by two reviewers (HK and MH) using the RoBANS tool [39]. The criteria for assessing the risk of bias were selection of participants, confounding variables, measurement of exposure, blinding of outcome assessment, incomplete outcome data, and selective outcome reporting.

### 4.5. Data Synthesis and Analysis

A standard meta-analysis was performed using Review Manager (RevMan, version 5.4; Cochrane Collaboration, Oxford, United Kingdom). The statistical heterogeneity between studies was evaluated using a chi-square test. Heterogeneity was defined as significant when the *p* value was <0.1 or the I^2^ value was >50%. Fixed and random effects models were applied when the data were considered homogenous and heterogeneous, respectively. The risk was calculated using ORs and 95% CIs. The pooled ORs and 95% CIs were calculated using a fixed- or random-effects model, and the ORs from these results were compared [37].

## 5. Conclusions

In conclusion, the present study revealed that ceftriaxone was comparable to sulbactam-ampicillin as an initial treatment of CAP in terms of clinical effectiveness. Therefore, our results support guidelines for the treatment of CAP, although more RCT studies are needed to obtain a deeper understanding.

## Figures and Tables

**Table 1:** Characteristics of the Studies Included in the Meta-Analysis.

**Study**	**Study Design**	**Setting**		**Period**	**Country of Study**	**No of Patients**		**Agent Used with Dosage**
Shinoda Y, 2016 [19]	Retrospective observational study	Single center		January 2013 to May 2013	Japan	SAM, 45; CRO, 9		SAM, NR; CRO, NR
Terafarad F, 2017 [20]	Retrospective observational study	Single center		January 2014 to December 2015	Japan	SAM, 20 CRO, 20		SAM, 3–6 g/day; CRO, 2–4 g/day
Hasegawa S, 2019 [21]	Retrospective observational study	Multicenter		September 2011 to August 2014	Japan	SAM, 400; CRO, 237		SAM, NR; CRO, NR
Hamao N, 2020 [22]	Open-label, randomized controlled trial	Single center		June 2002 to June 2008	Japan	SAM, 139; CRO, 124		SAM, 1.5–6.0 g/day; CRO, 1–2 g/day
**Study**	**Mean Duration of Therapy, Days**		**Mean Age, Years**		**Underlying Disease (%)**		**Pathogen (%)**	
Shinoda Y, 2016 [19]	SAM, NR; CRO, NR		Overall Over 80, 72.3%		Stroke, 45.5 Alzheimer’s disease, 33.7 Parkinson’s disease, 12.9		GPB, 9.9 GNB, 55.4	
Terahara F, 2017 [20]	SAM, NR; CRO, NR		SAM, 88; CRO, 81		Dementia, 55.0 Bedridden status, 30.0 Cerebrovascular disease, 27.5 Neuromuscular diseases, 7.5		NR	
Hasegawa S, 2019 [21]	SAM, NR; CRO, NR		SAM, 82; CRO, 82		Dementia, 28.9 Bedridden status, 16.3 Cerebrovascular disease, 4.4 Neuromuscular diseases, 11.9		Mainly *Streptococcus pneumoniae* and *Haemophilus influenzae*	
Hamao N, 2020 [22]	SAM, 7–14; CRO, 7–14		SAM, 63; CRO, 61		NR		*S. pneumoniae*, 23.2%; *H. influenzae*, 2.3%; unknown, 47.1%	

CRO, ceftriaxone; GNB, gram-negative bacteria; GPB, gram-positive bacteria; No., number; NR, not reported; SAM, sulbactam-ampicillin.

**Table 2:** Risk of bias in the included studies.

Study	Selection of Participants	Confounding Variables	Measurement of Exposure	Building of Outcome Assessment	Incomplete Outcome Data	Selective Outcome Reporting
Shinoda Y, 2016 [19]	Low risk	High risk	Low risk	High risk	Low risk	Low risk
Terahara F, 2017 [20]	Low risk	Low risk	Low risk	High risk	Low risk	Low risk
Hasegawa S, 2019 [21]	Low risk	Low risk	Low risk	High risk	Low risk	Low risk
Hamao N, 2020 [22]	Low risk	Low risk	Low risk	Low risk	Low risk	Low risk

## Data Availability

All data are applicable in the paper.

## Supplementary Materials

The following supporting information can be downloaded at: https://www.mdpi.com/article/10.3390/antibiotics11101291/s1, Figure S1: PRISMA 2020 Checklist. Refs. [40,41] are cited in Supplementary Materials.
